# Gastrointestinal Symptoms and Fermentable Oligosaccharides, Disaccharides, Monosaccharides, and Polyols Sources in Schoolchildren—A Pilot Study

**DOI:** 10.3390/children11060742

**Published:** 2024-06-18

**Authors:** Miguel Saps, Carlos Alberto Velasco-Benitez, Daniela Alejandra Velasco-Suarez, Maura Alvarez-Baumgartner, Amber N. Balda, Samantha Arrizabalo

**Affiliations:** 1 Department of Pediatrics, Division of Gastroenterology, Hepatology and Nutrition, Miller School of Medicine, University of Miami, Miami, FL 33136, USA; 2 Department of Pediatrics, Universidad del Valle, Cali 76001, Colombia

**Keywords:** schoolchildren, FODMAP, diet, abdominal pain, abdominal distention, nausea

## Abstract

Bothersome gastrointestinal (GI) signs/symptoms, including abdominal pain, distension, nausea, and flatulence, are common in children. A diet low in fermentable oligosaccharides, disaccharides, monosaccharides, and polyols (FODMAPs) is frequently recommended for children with GI symptoms. Currently, there are no studies on the effect of FODMAPs in healthy schoolchildren. In this cross-sectional study, schoolchildren reported an association between FODMAPs and GI symptoms through a standardized questionnaire and images of 20 common staples known to be rich in FODMAPs. A total of 208 schoolchildren aged 8–18 years old participated. A proportion of 38.0% of children reported GI symptoms, with abdominal pain (33%) being the most common complaint followed by abdominal distension (24%) and nausea (23%). The majority of children who reported intolerances to FODMAP-containing foods were intolerant to less than two food groups (76%). While vegetables and legumes (26%), particularly black beans (11%) and onions (7%), emerged as the most common group of triggers, milk (12%) stood out as the single food most frequently associated with GI symptoms. In conclusion, there was a high prevalence of FODMAPs intolerance among schoolchildren. Larger studies are recommended to confirm these findings and to inform possible dietary interventions to reduce the effect of FODMAPs on schoolchildren.

## 1. Introduction

Abdominal pain (AP) is a common problem in children worldwide [1,2,3,4,5]. In one study, thirty-nine percent of schoolchildren in Colombia reported abdominal pain weekly [6]. Schoolchildren with AP report that their pain interferes with daily activities including gym, sleep, and social outings. Abdominal pain in children is also associated with school absenteeism and parental work absences [1].

AP in children has been associated with dietary triggers (i.e., food allergies, intolerances) and various psychosocial events (i.e., stress, history of abuse, anxiety) [7,8]. The treatment of abdominal pain includes medication, psychosocial, and dietary interventions [9]. Most dietary interventions have not been thoroughly studied [10,11] in children. The most commonly prescribed diet for patients with abdominal pain, such as those with IBS, is a diet low in fermentable oligosaccharides, disaccharides, monosaccharides, and polyols (FODMAPs) [12,13]. FODMAPs are a group of foods high in short-chain carbohydrates that are poorly absorbed and may contribute to abdominal pain [14] in patients with visceral hypersensitivity [15]. Studies suggest a link between dietary modifications and symptom improvement in some patients [16]. However, the low-FODMAP diet is highly restrictive, difficult to adhere to, and associated with nutritional deficiencies [17]. This observation has led to the development of the “FODMAP gentle” concept, which prioritizes flexibility by keeping most foods on the menu and replacing only a limited number of high-FODMAP foods with lower-FODMAP alternatives [15,18].

The FODMAP content of a regular diet varies by country and culture. There are no studies on the content of FODMAPs in the diet of children of Latin America. Moreover, the type of FODMAPs associated with gastrointestinal symptoms in this group of children has not yet been studied. This limits the ability to introduce a gentle low-FODMAP diet, as the restriction of the local staple foods, which can be high in FODMAPs [19], may result in an inadvertently highly restrictive diet even in cases where it is intended to restrict only a few food groups. This study aims to assess the frequency of FODMAP intolerance among healthy schoolchildren.

## 2. Materials and Methods

A cross-sectional study was conducted to assess the prevalence of GI symptoms related to high-FODMAP food consumption among schoolchildren in Colombia.

Recruitment: Information about the study was distributed to the families of the schoolchildren (8 to 18 years) from two public schools, Ana de Jesús Romero and Julio Caicedo y Téllez, located in Cali and Palmira, Valle del Cauca, Colombia. Children whose parents consented and themselves assented to participate were included in the study. Parents completed a questionnaire on demographic information and past medical history. Children with organic diseases were excluded from the study.

A group of experienced local dieticians selected high-FODMAP foods commonly eaten by Colombian children. These FODMAP-containing foods were further categorized into five distinct groups: vegetables and legumes (high in GOS, fructan, and/or mannitol), fruits (high in fructose, mannitol, and/or sorbitol), wheat (high in fructan), dairy (high in lactose), and concentrated sweets such as jams and juices (high in fructose, sorbitol, and/or fructan). Children were shown 20 images of these foods and asked to select the ones associated with past experiences of GI symptoms such as abdominal pain, distension, nausea, fullness, flatulence, and diarrhea after consumption of each food. Participants were separated by age group: schoolchildren aged 8–12 and adolescents aged 13–18. In total, 806 children were invited to participate; after excluding for organic diseases (*n* = 31), incomplete questionnaires (*n* = 67), and refusal to participate, 208 children were included in the analysis.

This study was conducted between June 2022 and September 2022. It was approved by the Institutional Review Board (IRB) of Hospital Universitario del Valle “Evaristo Garcia” in Cali, Colombia, and by the academic authorities of each participating school.

Statistical Analysis: Data were analyzed through univariate and bivariate analyses when pertinent. Univariate analysis included measures of central tendency (percentage, mean, standard deviation, and range). The bivariate analysis included Student’s *t*-test, Fisher’s exact test, and chi-square test. Additionally, a *p*-value of less than 0.05 was considered statistically significant.

## 3. Results

The participants were predominantly of mixed race (51.0%); 66.8% were female, with a mean age of 14.2 years (SD = 2.1). Table 1 provides detailed information on the demographics for the participants (Table 1).

### 3.1. Symptom Prevalence and Association with FODMAPs

Among the 208 children who completed the questionnaires, 79 (38.0%) reported experiencing bothersome GI symptoms. Most of them reported symptom association with one or two FODMAP groups (28.85%), while a few (2.88%) reported an association of symptoms with four or five groups (Table 2). Of the 79 children who reported any GI symptoms associated with FODMAPs, 38 (48.1%) children associated their symptoms with only one FODMAP group and 22 (27.85%) reported association with two FODMAP groups. Only 24.06% of children who reported GI symptoms associated them with more than two FODMAP groups.

### 3.2. Common Symptoms and Their Frequency

Within the group experiencing symptoms, abdominal pain emerged as the most common complaint, affecting 69/79 participants (87.34%). This was followed by abdominal distention 51/79 (64.56%) and nausea 47/79 (59.49%) (Table 3).

### 3.3. Foods Implicated

**Figure 1:**
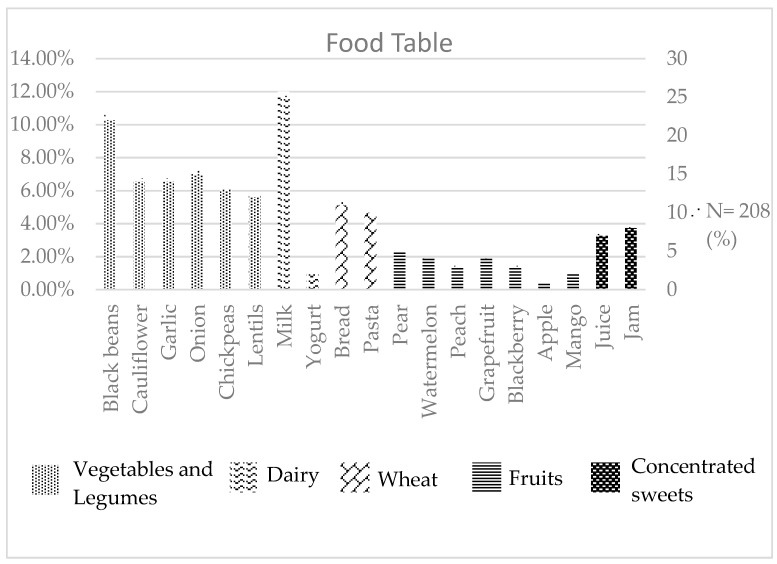
Food associated with GI symptoms (n = 208).

Children reported intolerances to common Colombian staples. Vegetables and legumes were identified as the primary FODMAP group associated with GI symptoms by 54 of the 79 (68.35%) children who reported any type of GI symptom. Onions, cauliflower, garlic, black beans, chickpeas, and lentils were the most frequently reported foods causing symptoms. Black beans were the most commonly reported intolerance within this group, followed by onions. Dairy was the second most common food group associated with GI symptoms. Milk specifically emerged as the single food item most frequently linked to these symptoms. Wheat foods like pasta and bread were also associated with symptoms, although less frequently compared to vegetables, legumes, and dairy. Fruits showed a weaker association with digestive discomfort. Out of 79 participants, 15 (18.99%) reported GI symptoms after consuming fruits. Pears were the most frequently reported fruits associated with discomfort, followed by watermelon and grapes. Among all groups, concentrated sweets such as jams and juices were the least reported group associated with symptoms (Table 4) (Figure 1).

## 4. Discussion

Our study found a high prevalence of FODMAP intolerance among the studied group. Almost 38% of participants experienced GI symptoms associated with FODMAPs intake. This suggests that FODMAP intolerance could be a contributor to the pathophysiology of abdominal pain, abdominal distention, nausea, fullness, flatulence, and diarrhea in schoolchildren. These findings highlight the need for further research exploring the potential benefits of a low-FODMAP diet as a management strategy for gastrointestinal symptoms in otherwise healthy children.

We found that vegetables and legumes were common triggers. Our study identified legumes (chickpeas, beans, and lentils) known for the high GOS content as a common source of GI symptoms in the studied group [20]. Participants reported the highest intolerance to black beans, followed by chickpeas and lentils, suggesting a specific pattern within legumes that warrants further investigation. A study by Tuck et al. (2018) exploring the link between IBS and GOS sensitivity found that adults with IBS who received alpha-galactosidase experienced a significant decrease in GI symptoms [21]. These findings suggest that GOS intolerance may contribute to abdominal pain, and managing GOS intake could be a potential strategy for reducing symptoms. In contrast to our study, an online survey conducted on Korean adults from the general population found a different result. The study found that GOS was the least common FODMAP type to trigger symptoms in both IBS and healthy individuals, however, the authors attribute this finding to GOS being rarely consumed by Korean people [22]. This suggests that the negative effect of specific FODMAPs may vary according to the dietary habits of each region, underscoring the importance of our investigation conducted in an understudied population.

While dairy products were the second most reported intolerance, milk itself was the most prevalent trigger of GI symptoms among our sample of schoolchildren (Figure 1). These findings are in agreement with the high prevalence of lactose intolerance reported in adults in Colombia [23]. Studies in children from Asia [24] and Europe have also shown high rates of lactose intolerance. A study from Switzerland found that milk was the most commonly avoided food by children due to GI symptoms [25].

Wheat-containing products are linked to GI symptoms in IBS patients [26]. In our study, wheat products emerged as the third most common dietary culprit associated with GI discomfort. A placebo-controlled study with cross-over rechallenge conducted in adults found no evidence of negative effects of gluten in patients with reported non-celiac gluten sensitivity when placed on a diet low in FODMAPs. This suggests the need for further research to better understand non-celiac gluten sensitivity and its relationship with FODMAPs [27,28].

Chumpitazi et al. found that fructans exacerbated symptoms in a selected group of children with IBS [29]. This was also reported in studies in adults with IBS [30,31]. Interestingly, our study conducted on a population of children who were not previously diagnosed with IBS, also found that fructan-rich vegetables, particularly onions and garlic, were prevalent food items associated with GI symptoms—in particular, abdominal pain, nausea, and flatulence. Among them, onions were reported most frequently by 15 (7.21%) participants, followed by garlic with 14 (6.73%).

Building on the concept of personalized diets, Bertin et al. (2024) proposed the “gentle FODMAP” approach for patients with IBS. This diet prioritizes the initial restriction of only the highest-FODMAP-containing foods under the guidance of a dietician [15]. This ultimately leads to a diet tailored to the specific needs of each patient [15,18,19]. As such, the “gentle diet” is thought to be “friendlier”, easier to adhere to, and less prone to producing nutritional deficiencies. Our study strengthens the case for the gentle FODMAP approach in children with GI symptoms. The majority (28.84%) of children reporting intolerance identified only one or two FODMAP groups as triggers. This suggests that a personalized, limited-restriction approach, rather than a very restrictive diet, may be suitable for children to improve their symptoms. Larger studies are needed to confirm these findings and establish personalized FODMAP restrictions as a potential dietary treatment for childhood abdominal pain.

Our study has multiple strengths and limitations. This is the first study to assess FODMAPs intolerance in schoolchildren. All pediatric studies on FODMAPs intolerance were conducted in patient consulting clinics, an approach prone to selection bias as only a small percentage of children who report abdominal pain seek medical care. A study by our group found that only 8.4% of all schoolchildren reporting abdominal pain in a school in Colombia visited a doctor for abdominal pain even though many reported limitations in daily function resulting from their symptoms [6]. The sample size of our study, although too small to assure generalizability, is the largest among all published studies of FODMAPs in children [32,33,34]. Our sample also included children of different races. This is an important strength, due to the possible effect of variable racial influences on the tolerance of specific food groups. In addition, a group of experienced local dieticians selected the food images presented to the children in effort to ensure that those staples were commonly consumed by children in the community.

Despite the strengths of our study, there are several limitations to consider. This includes the reliance on children’s self-reported data about symptoms following FODMAPs ingestion, which may introduce recall bias, especially in younger children who might have difficulty accurately recalling and reporting dietary intake and symptom severity. Additionally, the cross-sectional nature of the study limits the ability to ascertain long-term relationships between FODMAP intake and gastrointestinal symptoms. We recommend further research with larger sample sizes to explore the interplay between cultural dietary habits and individual FODMAP group tolerances across diverse populations and age groups. We recommend further research in larger multinational studies aimed at exploring the interplay between cultural dietary habits, individual FODMAP group tolerances, and how these factors vary across diverse populations and age groups to confirm our findings.

In conclusion, we found that a significant percentage of the children studied reported GI symptoms (38%) associated with the intake of foods containing high FODMAPs content. Most children reported intolerance to two or fewer FODMAP groups. This suggests that a personalized gentle approach restricting a small number of FODMAP food groups could potentially help improve gastrointestinal symptoms in healthy children. The data of this study and other similar studies could help the planning of school lunches.

## Figures and Tables

**Table 1:** Demographics.

	Gastrointestinal Symptoms (n = 208)		*p* Value
	No	Yes	
Age (Years)	n = 129	n = 79	
Mean (SD)	14.2 (2.0)	14.3 (2.2)	0.74
Age groups			
School age (8–12 years)	27 (20.9%)	14 (17.7%)	0.35
Adolescents (13–18 years)	102 (79.1%)	65 (82.3%)	
Sex			
Female	78 (60.5%)	61 (77.2%)	0.01
Male	51 (39.5%)	18 (22.8%)	
Race			
Mixed			
	64 (49.6%)	42 (53.2%)	0.67
White			
	36 (27.9%)	18 (22.8%)	0.51
Black			
	25 (19.4%)	17 (21.5%)	0.72
Indigenous			
	4 (3.1%)	2 (2.5%)	1

**Table 2:** Relation between FODMAP groups and GI symptoms (n = 208).

FODMAP Groups (n)	Children with GI Symptoms (n = 79)		All Children (n = 208)
1	38	48.1%	18.27%
2	22	27.85%	10.58%
3	13	16.46%	6.25%
4	3	3.8%	1.44%
5	3	3.8%	1.44%

**Table 3:** GI symptoms associated with FODMAP consumption.

GI Symptoms	Children with GI Symptoms (n = 79)		All Children (n = 208)
Any GI symptoms	79	100%	37.98%
Abdominal pain	69	87.34%	33.17%
Abdominal distention	51	64.56%	24.52%
Nausea	47	59.49%	22.6%
Fullness	46	58.23%	22.12%
Flatulence	36	45.57%	17.31%
Diarrhea	28	35.44%	13.46%

**Table 4:** Prevalence of FODMAP groups associated with GI symptoms.

Group of FODMAP	Children with GI Symptoms (n = 79)		All Children (n = 208)
Vegetables and Legumes	54	68.35%	25.96%
Dairy	25	31.65%	12.02%
Wheat	21	26.58%	10.1%
Fruits	15	18.99%	7.21%
Concentrated sweets	13	16.46%	6.25%

## Data Availability

The data presented in this study are available on request from the corresponding author. The data are not publicly available due to privacy and ethical restrictions.
